# Dietary Patterns Associated to Clinical Aspects in Crohn’s Disease Patients

**DOI:** 10.1038/s41598-020-64024-1

**Published:** 2020-04-27

**Authors:** Marina Moreira de Castro, Ligiana Pires Corona, Lívia Bitencourt Pascoal, Josiane Érica Miyamoto, Leticia Martins Ignacio-Souza, Maria de Lourdes Setsuko Ayrizono, Marcio Alberto Torsoni, Adriana Souza Torsoni, Raquel Franco Leal, Marciane Milanski

**Affiliations:** 1 School of Applied Sciences, University of Campinas (UNICAMP), Limeira, Brazil; 2 School of Medical Sciences, University of Campinas (UNICAMP), Campinas, Brazil

**Keywords:** Malnutrition, Crohn's disease, Nutrition

## Abstract

Diet is an important factor in both the pathogenesis and in the clinical course of Crohn’s disease (CD). However, data on dietary patterns of CD patients are rather limited in the literature. This cross-sectional study included 60 patients with CD, aged 18–60 years. Dietary intake was assessed using a validated food frequency questionnaire to measure food consumption patterns by principal component analysis (PCA). Multiple regression analysis was performed to investigate the association between dietary patterns and clinical and demographic variables. Three dietary patterns were identified: “Traditional + FODMAP” was associated with symptoms, gender, previous surgeries, and duration of the disease. “Fitness style” was positively associated with physical activity and negatively associated with body mass index and smoking. “Snacks and processed foods” was positively associated with duration of the disease and negatively associated with age. According to the weekly food consumption analysis, patients with active disease consumed less coffee and tea. We found significant associations between the three dietary patterns and the variables, but not with the stage of the disease. Prospective studies are necessary to determine the effects of food consumption patterns on the clinical course of CD.

## Introduction

Crohn’s disease (CD) causes chronic inflammation in the gastrointestinal tract^1^. Multiple factors play a role in the pathogenesis of inflammatory bowel disease (IBD), such as genetics, host immune dysregulation, gut microbiota and “Western style diet”^2,3^. This diet is known to be high in fats and calories, red and processed meat and n-6 polyunsaturated fatty acids (PUFA) and low in fruits and vegetables (for review see ref. ^4^). A case-control study in a Japanese population, in which the incidence of IBD is rapidly growing, found increased CD risk with higher consumption of sugars and sweeteners, sweets, fats and oils, and fish and shellfish^5^.

The role of diet has also been discussed in the clinical course of CD^6^. It is obviously difficult to perform interventional studies to analyze the role of diet in the clinical course of IBD. Analyses focused on the role of single nutrients or foods are the most common type of dietary study on CD. However, this approach is limited because nutrients and foods are not consumed in isolation, and investigating the significance of overall diet with focused attention on the dietary patterns approach represents an advance in nutrition that can facilitate nutritional recommendations, as well as the management of CD in order to induce and maintain the clinical and endoscopic remission of the disease^7^.

Diet patterns may also aggravate symptoms in patients with IBD. A Mexican study reported that foods such as beans, whole milk, plum, lima beans and spicy sauce increased the frequency of symptoms in patients with Ulcerative Colitis (UC), another condition of IBD^8^. In addition, a recent randomized controlled trial implemented within an Internet-based cohort found that the level of red and processed meat consumption does not reduce time for symptomatic relapse among CD patients to occur. However, this study presents significant limitations, as an online cohort was used to identify, recruit, enroll and follow up patients, and no direct contact was made with them^9^.

Therefore, the evaluation of the diet as a whole and the different dietary patterns defined by food groups should be taken into account. Data reported in the literature about the dietary patterns of patients with CD are scarce. Therefore, this study aimed to identify dietary patterns of patients with CD and to investigate the associated factors.

## Methods

### Study design and Study population

This cross-sectional study was performed on CD patients of the IBD outpatient clinic of the Coloproctology Unit at the Gastrocenter of the University of Campinas (UNICAMP) from May 2017 through July 2018. Patients were considered eligible for this study if they had a confirmed diagnosis of CD and had completed endoscopic or imaging tests within two months of recruitment. Only then were they invited to participate. The diagnosis of CD was based on endoscopic, radiological, and histological criteria. Inclusion criteria were: age between 18 and 60 years, with the disease located in the ileum and/or colon. Exclusion criteria were: pregnant and breastfeeding women, patients with edema and patients who lacked endoscopic or imaging tests.

This study protocol was approved by the Ethics Committee of the University of Campinas (CAAE n° 62802016.0.0000.5404) and a written informed consent form was obtained from all patients before the interview and data collection. Additionally, all methods were performed in accordance with the relevant guidelines and regulations.

### Dietary assessment

Dietary intake data were collected using a validated food frequency questionnaire (FFQ), which consisted of 76 food items in total with standard portion sizes, including foods rich in Fermentable Oligosaccharides, Disaccharides, Monosaccharides, and Polyols (FODMAP), such as apple, pear, watermelon, cauliflower, garlic, onion, milk, yogurt, etc. The frequency was evaluated in these categories: never or almost never; once a month; twice a month; once a week; twice or three times a week; four to five times a week; every day. Data were converted into an estimate of weekly consumption and the 76 food items were aggregated into 22 food groups, which were based according to the nutritional characteristics and the preliminary factor analysis.

### Statistical analysis

Principal component analysis (PCA) was used to identify food consumption patterns, a multivariate analysis using reported information to identify common underlying dimensions (factors or patterns) of food consumption, as it enables the items to be grouped based on the degree of correlation among them. The variables grouped in each factor are more strongly correlated with each other than with the variables belonging to the other factors. Therefore, this procedure enables the food items contained in the food consumption assessment instrument to be grouped on the basis of the degree of correlation among them. The interpretation and denomination of the factors, in the case of dietary patterns, depends on the meaning of each combination of the variables (food items) observed in the factor and, especially, those items with the highest factorial load^10^.

An eigenvalue cut-off>1 (which represents the total variance explained by each factor or diet component)^11^, and visual inspection scree plot chart were used to decide the number of components to retain. Afterwards, we performed an orthogonal rotation (varimax) to increase interpretability. Thus, we identified 3 factors. Factor loadings with values above 0.30 or lower than -0.3 were considered satisfactory so that the variable could become part of a pattern. The independent variables were: gender; age group (18–29 years, 30–60 years); CD stage (remission, activity); smoking; alcohol drinker; duration of the disease (<1 year, 1–5 years, 5–10 years, ≥10 years); number of frequent symptoms (none, 1–2, 3–4, ≥5); previous surgeries; body mass index (BMI) (low weight/eutrophic, overweight/obesity); physical activity. CD stage was evaluated by colonoscopy assessment (CDEIS is defined as 5 or the presence of deep ulcers in at least one intestinal segment) or by nuclear magnetic resonance scan enterography (presence of deep ulcers in at least one intestinal segment, as well as edema and creeping mesenteric fat near the affected intestinal area). After that, patients were divided into remission and activity groups.

Factors related to each pattern had their scores estimated and were described according to means and standard error, and the differences between groups was tested using the Student t test. Subsequently, univariate and multiple regression analysis using generalized linear models were made to investigate the association of each pattern with clinical and demographic variables. The significant variables or the ones that adjusted other variables <0.20 were maintained in multiple models. Data were analyzed using Stata® 14, with critical value of 5%.

### Ethical standards

This survey was approved by the Ethics Committee of the University of Campinas (62802016.0.0000.5404).

### Informed consent

All participants provided written informed consent to participate in this study.

## Results

A total of 60 patients with CD were interviewed. Of these, 31 patients were in remission and 29 in activity assessed by Crohn’s Disease Endoscopic Index of Severity (CDEIS). Most of the patients were male (51.7%), aged between 30 to 60 years (76.7%). We investigated if there were associations between these previously findings with dietary pattern. First, we analyzed the frequency of consumption and obtained three dietary patterns described in Table 1 as well as the percentage of variance explained for each factor. Pattern 1, named “traditional + FODMAP”, was mainly characterized by higher consumption of rice, pasta, red and poultry meat, legumes, industrialized juice, soft drink, and FODMAP. Pattern 2, named “fitness style” with high loadings for tapioca, eggs, pepper, olive, canned, and fruits. Pattern 3, called “Snacks and processed foods”, was mainly characterized by higher consumption of pizza, pie, snacks, cheese, red and poultry meat, and sausages. The three derived dietary patterns explained 38.4% of the variance in food intake.

**Table 1:** Distribution of factor loading of food/food groups in three food consumption patterns in CD patients.

Food/food groups	Patterns		
	Traditional + FODMAP	Fitness style	Snacks and processed foods
Rice, pasta	**0.427**	−0.062	−0.046
Breads, cookies	0.105	−0.001	0.090
Pizza, pie	−0.069	−0.028	**0.320**
Snacks	-0.102	−0.071	**0.474**
Tapioca	−0.039	**0.480**	−0.097
Sweets	0.095	−0.048	0.239
Lactose free milk, yogurt	−0.123	0.136	−0.154
Cheese	−0.040	0.010	**0.364**
Coffee, tea	0.112	−0.062	−0.018
Butter, margarine	0.205	−0.180	−0.143
Fish	0.149	−0.004	−0.169
Eggs	0.089	**0.468**	−0.056
Red, poultry meat	**0.302**	−0.016	**0.300**
Sausages	0.081	−0.018	**0.442**
Pepper	−0.003	**0.332**	−0.049
Olive, canned	−0.100	**0.345**	0.200
Vegetables	0.202	0.151	0.183
Fruits	−0.008	**0.329**	0.023
Legumes	**0.439**	−0.123	−0.070
Industrialized juice, soft drink	**0.404**	0.189	0.017
Seasoning	0.217	−0.127	−0.120
FODMAP*	**0.354**	0.230	0.052
Explained Variance by factor (%)	15.1	12.3	10.9
Accumulated variance (%)	15.1	27.5	38.4

*FODMAP: Fermentable Oligosaccharides, Disaccharides, Monosaccharides, and Polyols.

Table 2 displays the mean scores of food patterns according to independent variables. The scores of the pattern “traditional + FODMAP” were significantly higher in males and patients with ≥5 symptoms. The scores of the pattern “snacks and processed foods” were significantly higher in males and patients aged from 18 to 29 years. We did not observe significantly higher scores in the pattern “fitness style”.

**Table 2:** Mean scores (standard error) of food consumption patterns according to clinical and demographic characteristics of CD patients.

Variables			Patterns	
	*n*, %	Traditional + FODMAP	Fitness style	Snacks and processed foods
Gender		**p** = **0.024**	p = 0.799	**p** = **0.032**
Male	31, 51.7	0.508 (0.388)	0.052 (0.368)	0.410 (0.321)
Female	29, 48.3	−0.543 (0.220)	−0.056 (0.205)	−0.438 (0.207)
Age group (years)	14, 23.3	p = 0.592	p = 0.472	**p** = **0.031**
18 to 29	46, 76.7	0.231 (0.684)	−0.280 (0.310)	0.774 (0.572)
30 to 60	31, 51.7	−0.070 (0.230)	0.085 (0.261)	−0.235 (0.185)
CD stage	29, 48.3	p = 0.654	p = 0.535	p = 0.894
Remission		−0.103 (0.280)	−0.128 (0.204)	0.026 (0.253)
Activity		0.110 (0.388)	0.137 (0.385)	−0.027 (0.317)
Smoking		p = 0.266	p = 0.263	p = 0.888
Yes	1, 1.7	−2.007 (−)	−1.826 (−)	−0.218 (−)
No	59, 98.3	0.034 (0.237)	0.030 (0.214)	0.003 (0.203)
Alcohol drinker		p = 0.380	p = 0.980	p = 0.847
Yes	22, 36.7	0.274 (0.468)	0.007 (0.231)	0.051 (0.250)
No	38, 63.3	−0.158 (0.256)	−0.004 (0.310)	−0.029 (0.282)
Duration of the disease (years)		p = 0.161	p = 0.753	p = 0.176
<1	5, 8.3	−0.998 (0.397)	0.216 (0.620)	−0.913 (0.415)
1 to 5	15, 25	0.333 (0.670)	−0.054 (0.285)	−0.049 (0.341)
5 to 10	12, 20	−0.106 (0.389)	−0.484 (0.266)	0.180 (0.400)
≥10	28, 46.7	0.045 (0.308)	0.198 (0.401)	0.112 (0.340)
Number of frequent symptoms		**p** = **0.017**	p = 0.623	p = 0.125
None	20, 33.3	0.039 (0.281)	−0.075 (0.229)	0.509 (0.467)
1 to 2	28, 46.7	−0.085 (0.308)	−0.006 (0.405)	−0.182 (0.222)
3 to 4	10, 16.7	−0.471 (0.533)	0.062 (0.353)	−0.366 (0.333)
≥5	2, 3.3	3.159 (4.807)	0.538 (1.422)	−0.705 (1.147)
Previous surgeries		p = 0.061	p = 0.878	p = 0.410
Yes	44, 73.3	0.265 (0.292)	0.019 (0.275)	−0.100 (0.239)
No	16, 26.7	−0.729 (0.306)	−0.054 (0.262)	0.275 (0.362)
BMI (kg/m^2^)		p = 0.774	p = 0.112	p = 0.992
Low weight/Eutrophic	35, 58.3	0.057 (0.332)	0.286 (0.333)	0.001 (0.209)
Overweight/Obesity	25, 41.7	−0.081 (0.328)	−0.400 (0.189)	−0.002 (0.386)
Physical Activity		p = 0.616	p = 0.078	p = 0.460
Yes	15, 25	0.206 (0.427)	0.648 (0.690)	0.258 (0.513)
No	45, 75	−0.068 (0.281)	−0.216 (0.161)	−0.086 (0.207)

FODMAP: Fermentable Oligosaccharides, Disaccharides, Monosaccharides, and Polyols.

**Figure 1:**
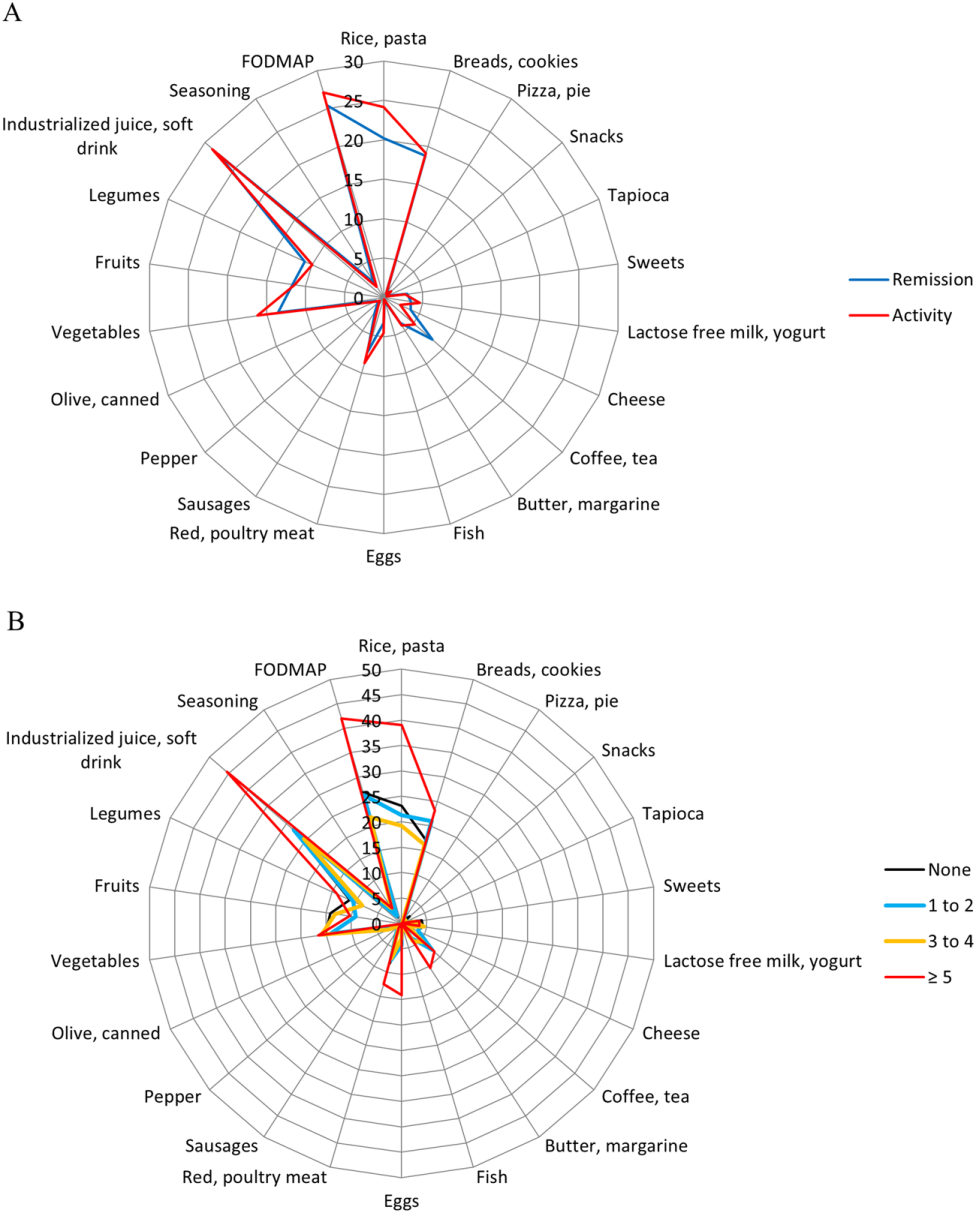
Detailed description of the 22 foods groups used in the PCA. Food groups distribution according to remission and activity CD (**A**). Food groups distribution according to patients’ symptoms in both remission and activity CD (**B**). PCA: principal component analysis. FODMAP: Fermentable Oligosaccharides, Disaccharides, Monosaccharides, and Polyols.

No difference was observed in dietary patterns between remission and active disease group, so we chose to present a qualitative analysis of mean weekly consumption of each food group according to disease phase (Fig. 1). The reason for this is that when we observe the spatial representation of consumption of food portions distribution by patients in active disease or in remission, the pattern formed is similar (Fig. 1A). However, the consumption of coffee and tea was lower in the activity group than in the remission group (p = 0.015). When we observed in more detail the consumption in portions, in relation to patient’s symptoms, the pattern formed is also similar between the groups, with the exception for some food group distribution in relation to patients with ≥5 symptoms, such as rice, pasta, breads, cookies, butter, margarine, eggs, red and poultry meat, industrialized juice, soft drink, and FODMAP (Fig. 1B).

Concerning the three identified dietary patterns, we analyzed the associations with the studied variables. As observed in Table 3, the factors associated with the pattern “traditional + FODMAP” were symptoms, gender, previous surgeries, and duration of the disease. The pattern “fitness style” was positively associated with physical activity, but negatively associated with BMI and smoking. The pattern “snacks and processed foods” showed negative association with age and positive association with duration of the disease (>10 years).

**Table 3:** Analysis of multiple linear regression of clinical and demographic factors associated with food consumption patterns in CD patients.

Variables	β	95% CI	*p* value
Pattern “Traditional + FODMAP”			
Number of frequent symptoms			
≥5	3.456	(0.924, 5.988)	0.007
Gender			
Male	−1.192	(−2.100, −0.283)	0.010
Previous surgeries	1.092	(0.073, 2.111)	0.036
Duration of the disease			
1 to 5	1.758	(0.068, 3.448)	0.041
Pattern “Fitness style”			
Physical Activity	1.632	(0.654, 2.611)	0.001
BMI (kg/m^2^)			
Overweight/Obesity	−1.454	(−2.318, −0.590)	0.001
Smoking	−3.795	(−6.881, −0.708)	0.016
Pattern “Snacks and processed foods”			
Age			
30 to 60	−1.354	(−2.317 −0.390)	0.006
Duration of the disease			
>10	1.470	(0.029, 2.912)	0.045

FODMAP: Fermentable Oligosaccharides, Disaccharides, Monosaccharides, and Polyols.

## Discussion

As it is difficult to assess the effect of isolated foods group in the clinical course of patients with IBD, we evaluated the association between the dietary patterns and its related factors in both remission and activity CD outpatients. A dietary pattern is a comprehensive food evaluation in which multiple foods and nutrients are studied collectively. This approach is a simplification designed to make it easier to understand complex diet-disease relations^12^. Identification of dietary patterns is based on the correlations in eating habits, making this analysis a more powerful and valid measure of food intake^13^.

Our results reveal important associations between the three identified dietary patterns and their associated factors, as well as complex combinations of food intake. Pattern 1 (traditional + FODMAP) which, as expected, explained the greater variance among the factors (15.1%), reflects traditional Brazilian eating habits and is characterized mainly by the consumption of rice and legumes, especially beans. This pattern was associated with ≥5 symptoms, previous surgeries, and 1 to 5 years of the disease and negatively associated with being male. It is possible that differences between men and women occur in relation to diet in CD, and investigations are necessary to verify a possible role of sex in diet-related course of CD. Flood *et al*. found that differences were observed between sex and dietary pattern in patients with colorectal cancer, and the authors suggest a possible role of sex in disease etiology^14^.

The positive association of Pattern 1 (traditional + FODMAP) with previous surgeries in our study may be explained by the improvements in the BMI parameter, and in food intake in the postoperative period. The state of well-being after recovery from surgery may lead to the less selective food intake, including FODMAPs, which could in turn lead to the appearance of abdominal symptoms, even in the absence of active endoscopic disease. The recovering of the nutritional status after surgery depends on the total length of the resected bowel and the adopted technique (resection and/or stricturoplasty). Patients who have undergone only intestinal stricturoplasty, a conservative surgical technique often used in CD treatment, frequently cease to have obstructive symptoms and also gain weight secondary to oral intake improvement^15^. Moreover, in the majority of patients from our study who underwent intestinal resection, the maximum resected intestinal length was up to 40 cm, which did not affect the intestinal absorption and the food-intake, and improved the quality of life after surgery. Up to 80% of CD patients undergo at least one surgery in their lifetime, which is commonly required for patients with complications or when medical management is unsuccessful^16^. The consumption of FODMAP, observed in this pattern, has been studied with an emphasis on the low FODMAP diet in IBD patients presenting Irritable Bowel Syndrome (IBS)-like symptoms, showing significant improvement in the symptoms^17,18^. FODMAPs are poorly absorbed in the small intestine and subsequently fermented by bacteria in the colon, resulting in abdominal distension that can trigger symptoms in sensitive individuals, such as abdominal pain, bloating, diarrhea and/or constipation, and flatulence^19,20^. A randomized controlled trial investigated quiescent IBD patients following a low FODMAP diet, and found improvements in symptom relief and a lower abundance of fecal microbes believed to regulate the immune response^21^. However, this diet presents short and long-term limitations, such as high level of food restriction, which results in low fiber and micronutrient intakes, which in turn could lead to undesirable changes in gut microbiota composition^22^.

Pattern 2 was named “fitness” because the food items that compose it are usually part of diets adopted by more health-conscious people, and are also more susceptible to “fashion” diets. Tapioca is a starch extracted from the storage roots of the cassava plant, highly consumed in the north region and central-west region of Brazil^23^. Lately, physically active persons are adopting gluten-free-based diets to replace traditional carbohydrate-based flour-based cereals, so tapioca has become very popular because of its healthy, gluten-free claim in other regions of Brazil, mainly in the south east, where this research was conducted. Besides, other food items like olives, pepper and eggs are being considered healthy for some part of the population, for their functional properties, disseminated mainly in non-scientific publications and social media. Although a gluten-free diet could benefit some CD patients, it is important to identify this kind of eating behavior when is tending to become extreme, to provide correct information and to clarify the role of a healthy diet based on nutritional guidelines, not on dietary fads.

Reinforcing this hypothesis, “fitness style” pattern was positively associated with physical activity, in which a meta-analysis showed that it has a protective effect in CD^24^; whereas higher BMI and smoking were negatively associated with this pattern.

Pattern 3 (snacks and processed foods) was associated with>10 years of the disease and negatively associated with older age group, between 30 and 60 years. This result is often reported, as younger patients tend to have higher intakes of snacks and processed foods. According to the latest Brazilian household food budget survey, sweets, milk-based flavored drinks and cookies, for example, appeared among the most commonly consumed foods only for adolescents and young adults^25^. Probably, patients with longer disease duration have a less restricted dietary intake and consequently the processed foods consumption is more common. Moreover, a study demonstrated that disease duration was associated with a less severe course^26^. The knowledge about the long-term evolution of CD is not well-known. However, a higher proportion of patients achieved remission with more than 15 years of diagnosis in a regional cohort of CD patients^27^, supporting the finding that CD activity declines over time. As previously shown by our group^28^, the mean duration of the disease in patients in remission (12.64 years) was higher than in patients in activity (8.27 years), as well as the mean age (remission 39.67 years vs activity 33.82 years).

We did not find any associations between eating patterns and the stage of the disease (remission and activity). This result is consistent with some previous studies, as we describe below, even if comparison between the studies is difficult because of the differences in dietary pattern composition identified as well as the high variability in the population.

The study by Taylor *et al*. explored and compared the dietary pattern of CD patients in remission with the Mediterranean diet according to the Mediterranean diet scores (P-MDS) recommendations in a Canadian ambulatory clinic. They identified low intakes of olive oil, legumes, nuts, fruits and vegetables. Only a few patients met the P-MDS criteria for intake of vegetables or legumes, the median intakes of fruit were 50% below the recommendations, 30% of men exceeded red or processed meats recommendations, and over 80% of patients reported inadequate intake of fish and nuts^29^. In our study, as we could not identify differences in dietary pattern between remission and activity groups, we explored some isolated food groups, and observed lower consumption of coffee and tea among patients with active disease. Barthel *et al*. showed that patients with CD attributed negative effects of coffee intake in their course of the disease^30^. Besides, the stimulatory effects of coffee or its caffeine content on gastrointestinal motility might increase stool frequency and consequently adversely affect IBD symptoms^31^.

In the recent literature, restricted diets, such as the Specific Carbohydrate Diet (SCD)^32,33^, the Anti-Inflammatory Diet (IBD-AID)^34^ and Low FODMAP diet^18,20,35^ have presented promising effects on reducing symptoms and have been suggested for patients with IBD, which highlights the increasing interest in performing interventional studies in CD. Besides that, CD Exclusion Diet, coupled with partial enteral nutrition (CDED plus PEN)^36^ and a food-based diet (CD-TREAT), demonstrated potential to induce sustained remission and reduce gut inflammation^37^. In an interesting internet-based cohort study, it was shown that patients in activity reported different dietary patterns from those of patients in remission, as a perception of the influence of food in their disease course. The survey also documented that rice was frequently reported to improve symptoms, whereas vegetables, fruits, high-fiber fruits, and soda were more frequently reported to worsen symptoms in CD patients^38^. Our findings suggest that in general the patients do not undergo counselling by a nutritionist, or if they do, may not be able to follow their recommendations. Accordingly, nutritional screening is essential as part of IBD patients’ health care in order to minimize the nutritional disorders and consequently, to help to improve the dietetic therapy and the quality of life, as presented in ESPEN guideline^39^. The influence of diet in the pathogenesis of CD is usually recognized; however, there are limited guidelines and original data demonstrating the positive effects of specific dietary counselling for the management and during the course of CD^40^.

The predictors of relapse, which may include the environmental factors, could represent an important advance in clinical management and in the future therapeutic strategies^41^. Recently, the association between the course of the disease and dietary intake was investigated in a study of 103 adult UC patients. Tasson *et al*. found a significant inverse association between legumes and potato intake and the risk of relapse (adjusted Odds Ratio (OR) 0.21), suggesting a possible protective role in maintaining patients in remission. A detrimental role of meat, which corresponded to a higher risk of active disease (unadjusted OR 3.61), was also detected^42^.

This study has some limitations that should be addressed. First, it is an observational design study and we cannot determine causality. Thus, interventional studies are necessary to evaluate the influence of diet on the course of CD and the effects of food consumption patterns on disease severity in order to make recommendations on diet modifications. However, dietary intervention studies are considered complex, taking into account their challenges, including the recruitment and retention of patients and the economic costs^43^. It is important to highlight that when the disease activity was assessed we considered the last two months, while the usual dietetic assessment (FFQ) was referred to the last six months. Some aspects that could influence the results were not addressed in this study, for example the assessment of physical activity was only self-informed and we did not control for former alcohol drinkers, usually a confounding group. Finally, since this is a cross-sectional study, it is not possible to test cause-effect relations.

Furthermore, our sample size is somewhat restricted to the use of pattern analysis, and therefore the food groups had to be aggregated for analysis to be feasible. Nevertheless, the patterns extracted from the factor analysis accounted for 38.4% of the total variance in food intakes. Considering previous studies with the same analysis, this percentage is relatively high. Myklebust-Hansen *et al*. explained 10.98% of the variance in 3 factors in the study of IBD women^44^. Thus, the findings concerning the food consumption pattern and the food groups presented here can be considered good indicators of the habits of the CD patients.

The combination of nutritional education and dietary modification improves nutritional awareness, making it an effective approach for potentially promoting health^43,45^. In addition, the increasing prevalence of IBD is a contributor to the growth of public healthcare costs. Therefore, this study emphasizes the need for future studies targeting a dietary intervention in CD outpatients in order to optimize dietary intake and to improve their quality of life.

In summary, we identified three different dietary patterns among CD patients as well as their association with important clinical and demographic variables. Pattern 1 *“traditional* + *FODMAP”* was associated with ≥5 symptoms, previous surgeries, and 1 to 5 years of the disease and negatively associated with male; pattern 2 *“fitness style”* was positively associated with physical activity and negatively associated with BMI and smoking and pattern 3 *“snacks and processed foods”* was associated with>10 years of the disease and negatively associated with older age group, between 30 and 60 years. Although this study has not demonstrated associations between eating patterns and the stage of the disease, we noted a lower intake of coffee and tea during the active phase of CD. Moreover, more research should be performed in the near future to explore the benefits of nutritional treatment so as to improve the quality of food consumption and its effects on clinical outcomes, which could lead to a great public health impact in this specific population.

## Supplementary information

Table S1.

## Data Availability

Please contact the corresponding author for data requests.
