# Supplementary material for: Dietary Patterns Associated to Clinical Aspects in Crohn’s Disease Patients
Source: Sci Rep. 2020 Apr 27;10:7033. doi: 10.1038/s41598-020-64024-1 (PMC7184619; doi:10.1038/s41598-020-64024-1)
Supplement: Supplementary file 1 — Table S1. [file 41598_2020_64024_MOESM1_ESM.docx]

**Dietary Patterns Associated to Clinical Aspects in Crohn’s Disease Patients**

Marina Moreira de Castro^1,2^*****, Ligiana Pires Corona^1,2^*****, Lívia Bitencourt Pascoal^2^, Josiane Érica Miyamoto^1^, Leticia Martins Ignacio-Souza^1^, Maria de Lourdes Setsuko Ayrizono^2^, Marcio Alberto Torsoni^1^, Adriana Souza Torsoni^1^, Raquel Franco Leal^2^, Marciane Milanski^1,2^

^1^School of Applied Sciences, University of Campinas (UNICAMP), Limeira, Brazil

^2^School of Medical Sciences. University of Campinas (UNICAMP), Campinas, Brazil

***Corresponding author**

Marciane Milanski: marciane.milanski@fca.unicamp.br

**Table S1**

| **Food** | **Daily** | **Weekly** | **Monthly** | **Never/less than once/month** | **Food** | **Daily** | **Weekly** | **Monthly** | **Never/less than once/month** |
| --- | --- | --- | --- | --- | --- | --- | --- | --- | --- |
| Rice^a^ ( ) White  ( ) brown |  |  |  |  | Eggplant^k^ |  |  |  |  |
| Bread^b^ |  |  |  |  | Ginger^k^ |  |  |  |  |
| Pizza, pie^c^ |  |  |  |  | Pod^k^ |  |  |  |  |
| Sandwiches^d^ |  |  |  |  | Lettuce^k^ |  |  |  |  |
| Snacks^d^ |  |  |  |  | Olives^k^ |  |  |  |  |
| Pasta^e^ |  |  |  |  | Potato^k^ |  |  |  |  |
| Cookies^f^ |  |  |  |  | Spinach^k^ |  |  |  |  |
| Tapioca^g^ |  |  |  |  | Tomato^k^ |  |  |  |  |
| Candies^h^ |  |  |  |  | Zucchini^k^ |  |  |  |  |
| Milk^i^ ( ) regular  ( ) lactose free |  |  |  |  | Fruits |  |  |  |  |
| Yogurt^d^ ( ) regular  ( ) lactose free |  |  |  |  | Apple^m^ |  |  |  |  |
| White cheese^j^ |  |  |  |  | Apricot^m^ |  |  |  |  |
| Yellow cheese^j^ |  |  |  |  | Cherry^m^ |  |  |  |  |
| Coffee and tea^i^ |  |  |  |  | Blackberry^m^ |  |  |  |  |
| Alcoholic drinks^i^ |  |  |  |  | Mango^m^ |  |  |  |  |
| Margarine^k^ |  |  |  |  | Nectarine^m^ |  |  |  |  |
| Butter^k^ |  |  |  |  | Peach^m^ |  |  |  |  |
| Olive oil^k^ |  |  |  |  | Pear^m^ |  |  |  |  |
| Fish^l^ |  |  |  |  | Persimmon^m^ |  |  |  |  |
| Eggs^d^ |  |  |  |  | Plum^m^ |  |  |  |  |
| Beef^l^ |  |  |  |  | Watermelon^m^ |  |  |  |  |
| Chicken^l^ |  |  |  |  | Banana^m^ |  |  |  |  |
| Pork^l^ |  |  |  |  | Blueberry^m^ |  |  |  |  |
| Sausages^d^ |  |  |  |  | Grape^m^ |  |  |  |  |
| Raw vegetables |  |  |  |  | Lemon^m^ |  |  |  |  |
| Cooked vegetables^k^ |  |  |  |  | Orange^m^ |  |  |  |  |
| Cauliflower^k^ |  |  |  |  | Passion fruit^m^ |  |  |  |  |
| Broccoli^k^ |  |  |  |  | Raspberry^m^ |  |  |  |  |
| Artichokes^k^ |  |  |  |  | Strawberry^m^ |  |  |  |  |
| Asparagus^k^ |  |  |  |  | Nuts and seeds^k^ |  |  |  |  |
| Garlic^k^ |  |  |  |  | Textured soy protein^i^ |  |  |  |  |
| Mushrooms^k^ |  |  |  |  | Legume^a^ |  |  |  |  |
| Onion^k^ |  |  |  |  | Canned food^k^ |  |  |  |  |
| Scallion^k^ |  |  |  |  | Juices^n^ ( ) 100% ( ) industrialized |  |  |  |  |
| Carrot^k^ |  |  |  |  | Soda^n^ |  |  |  |  |
| Pepper^k^ |  |  |  |  | Seasoning^k^ |  |  |  |  |
| Cucumber^k^ |  |  |  |  | Others:  Fried foods |  |  |  |  |

^a^Equivalent to 0.5 cup

^b^Equivalent to ½ unit / 1 slice

^c^Equivalent to 1 average slice

^d^Equivalent to 1 unit

^e^Equivalent to 0.5 cup / 1 average slice / 1 unit

^f^Equivalent to 5 cookies

^g^Equivalent to 4 tablespoons

^h^Equivalent to 1 tablespoon / 1 slice

^i^Equivalent to 1 cup

^j^Equivalent to 1 oz cheese

^k^Equivalent to 1 tablespoon

^l^Equivalent to 4 oz beef, chicken, pork or fish

^m^Equivalent to 1 average slice / 1 unit / 1 cup

^n^Equivalent to 1.25 cup
